# Changes to aspects of ongoing randomised controlled trials with fixed designs

**DOI:** 10.1186/s13063-020-04374-3

**Published:** 2020-06-03

**Authors:** Xanthi Coskinas, John Simes, Manjula Schou, Andrew James Martin

**Affiliations:** 1 The National Health and Medical Research Council Clinical Trial Centre, University of Sydney, Camperdown, NSW 2050 Australia; 2 Department of Mathematics and Statistics, Macquarie University, Macquarie Park, NSW Australia; 3 Janssen-Cilag Pty. Limited, Macquarie Park, NSW Australia

**Keywords:** Randomised control trials, Bias, Design changes, Type 1 error

## Abstract

**Background:**

Despite careful planning, changes to some aspects of an ongoing randomised clinical trial (RCT), with a fixed design, may be warranted. We sought to elucidate the distinction between legitimate versus illegitimate changes to serve as a guide for less experienced clinical trialists and other stakeholders.

**Methods:**

Using data from a large trial of statin therapy for secondary prevention, we generated a set of simulated trial datasets under the null hypothesis (H0) and a set under an alternative hypothesis (H1). Through analysis of these simulated trials, we assessed the performance of the strategy of changing aspects of the design/analysis with knowledge of treatment allocation (illegitimate) versus the strategy of making changes without knowledge of treatment allocation (legitimate). Performance was assessed using the type 1 error, as well as measures of absolute and relative bias in the treatment effect.

**Results:**

Illegitimate changes led to a relative bias of 61% under H1, and a type 1 error rate under H0 of 23%—well in excess of the 5% significance level targeted. Legitimate changes produced unbiased estimates under H1 and did not inflate the type 1 error rate under H0.

**Conclusions:**

Changes to pre-specified aspects of the design and analysis of an ongoing RCT may be a necessary response to unforeseen circumstances. Such changes risk introducing a bias if undertaken with knowledge of treatment allocation. Legitimate changes need to be adequately documented to provide assurance to all stakeholders of their validity.

## Background

Despite the emergence of adaptive trial designs such as multi-arm multi-stage (MAMS) designs [1] and adaptive Bayesian designs [2], the standard phase III randomised controlled trial (RCT) framework characterised by the pre-specification of fixed design elements remains the mainstay of clinical research. Despite careful prospective planning, changes to some aspect of an ongoing RCT, with a fixed design, may be warranted in reaction to new information arising. The new information triggering a *reactive revision* could potentially come from a variety of sources, including: another recently completed RCT investigating a similar question; basic research revealing new insights into the pathophysiology of a disease; and/or a clinical study clarifying the pharmacokinetic/pharmacodynamic properties of an experimental treatment. Reactive revisions to the planned analysis approach may arise as a consequence of seeking greater statistical power (to correctly reject the null hypothesis) and/or to accommodate unexpected characteristics of the trial data. Some examples of reactive revisions to the primary endpoint, the analysis set composition and the analysis method are presented in Table 1.

**Table 1:** Examples of reactive revisions

1. A reactive revision to the choice of primary endpoint occurred in the FIELD trial. The FIELD trial randomised 9795 patients with type 2 diabetes mellitus to fenofibrate (a drug that modifies the lipid profile) or placebo [3]. The original primary endpoint of coronary heart disease (CHD) death was changed to ‘any coronary event’ on the basis of a blinded (pooled) review of interim data that indicated a lower than expected CHD death rate that was inadequate for reaching the statistical power target.	
2. A reactive revision to the analysis set composition arose in the CO17 randomised controlled trial of cetuximab in patients with pre-treated metastatic epidermal growth factor receptor (EGFR)-positive colorectal carcinoma in response to external evidence suggesting that K-ras wild-type was a predictive biomarker. The effect of cetuximab was indeed found to be largely restricted to this subset of randomised patients [4, 5].	
3.The Sentinel Node Biopsy versus Axillary Clearance (SNAC1) trial changed the primary analysis from a comparison of proportions (of patients experiencing lymphoedema) to a comparison of means (of arm swelling) to take advantage of the greater statistical power of parametric analysis methods [6].	

Even when no reactive revision is made to aspects of an RCT, there may remain opportunities for flexibility in the form of *discretionary* decisions relating to analysis details that were not specified in the original protocol. Examples could include: whether or not to exclude from the analysis set a small proportion of patients found (after randomisation) to have been ineligible (on the basis of pre-randomisation factors); refinement of an ambiguous endpoint definition [7]; whether or not to adjust for a particular baseline covariate; or the choice of statistical test to compare randomised groups on a given endpoint.

Reactive revisions and discretionary decisions represent mechanisms for changing design and/or analysis aspects of an ongoing RCT, and such changes may bias results. The potential for bias rests on whether the change is based on information that is completely independent of treatment allocation, as opposed to information that is related to treatment allocation. Changes should be regarded as legitimate if the former can be assured and the revision is otherwise sound (i.e. the revised question is still regarded as important, the revised design has adequate power, the study remains ethical, etc.).

The fundamental distinction between legitimate versus illegitimate changes is no doubt well understood by experienced trialists, biostatisticians and clinical epidemiologists. Evidence of illegitimate changes nevertheless arising in the literature [8–16] suggests that there is value in illustrating the distinction to a broader constituency of stakeholders in clinical trials research to help ensure that: less experienced trialists do not inadvertently introduce (the perception of) a bias to their RCT; and reviewers/peers do not incorrectly judge legitimate changes as threats to the credibility of a well-conduced RCT. We sought to achieve this via a simulation study following the framework of Morris et al. [17].

### Aim

This study aimed to investigate the effect of allowing various changes to the planned design/analysis of an RCT, with and without knowledge of treatment allocation, under a scenario where there is no true difference between treatment groups (H0, null hypothesis) and when there is a true difference between treatment groups (H1, alternative hypothesis).

## Methods

### The Long-term Intervention with Pravastatin in Ischemic Disease (LIPID) trial

We chose to undertake the simulation study using data from a real RCT, rather than completely simulating the data, because this avoided the need to make assumptions about the joint probability distribution of patient baseline characteristics and outcomes. We used data from the LIPID trial that randomised—to pravastatin (a drug that modifies the lipid profile) or to placebo—a total of 9014 patients who had experienced an acute myocardial infarction (AMI) or a hospital discharge diagnosis of unstable angina pectoris (UAP) in the preceding 3–36 months [18]. Patients were recruited from 87 sites and were followed over a median of 6 years. The primary analysis for LIPID specified: time to CHD death as the primary endpoint; all randomised patients as the analysis set; and no adjustment for any baseline patient characteristics [18]. Cox proportional hazards regression was used to express the treatment effect as a hazard ratio (HR).

### Data-generating mechanisms

We constructed 100,000 simulated LIPID trial datasets under the null hypothesis (H0), where a HR of 1 was imposed (reflecting no difference in the hazard of CHD death between experimental and control treatment groups), and 100,000 simulated LIPID trial datasets under an alternative hypothesis (H1), where a HR of 0.85 was imposed (i.e. the hazard of CHD death was 15% lower in the experimental group compared to the control group). Full details of how this was done are presented in Additional file 1. All simulated trials were analysed using Cox proportional hazards regression, where any one of 72 unique analysis configurations could be applied to the analysis of an individual simulated trial. The configurations were constructed via a combination of options presented in Table 2 relating to: the choice of endpoint (three options); the analysis set composition (three options); and covariate specification (eight options). More information on these analysis configurations is presented in Additional file 1. Each configuration was considered a methodologically reasonable option—we did not investigate options with a known potential to introduce a bias, such as per-protocol type analyses that exclude patients with poor adherence to protocol therapy.

**Table 2:** Analysis configurations arising from three decision categories

Type of decision	Decision options
Choice of endpoint	1. Coronary heart disease (CHD) death^a^ 2. Revascularisation 3. All-cause mortality
Analysis set composition	1. No exclusions (i.e. all randomised patients)^a^ 2. Exclude patients with eGFR < 45 (Stage 3a kidney disease or worse) (excludes 4%) 3. Exclude patients with qualifying events within 9 months of randomisation (excludes 29%)
Covariate specification	1. None^a^ 2. Stroke (2 levels: yes, no) 3. Smoking status (2 levels: never smoked, current/ex-smoker) 4. Index ACS (3 levels: unstable angina pectoris (UAP), single myocardial infarction (MI), multiple MI) 5. Stroke and smoke 6. Stroke and index ACS 7. Smoke and index ACS 8. Stroke, smoke and index ACS

*ACS* acute coronary syndrome, *eGFR* estimated glomerular filtration rate

^a^Base-case analysis: CHD death, no exclusions, no baseline covariate adjustment

### Targets and analysis methods

The targets for our simulation studies were the log hazard ratio (*θ*) and type I error. We used the coefficient ($\hat{\theta}$) for treatment obtained from a Cox proportional hazards model as the estimate for the estimand (*θ*), and used the Wald test associated with $\hat{\theta}$ to produce a *p*-value.

### Performance measures

We estimated the following:

- the type I error rate using $\frac{1}{n_{sim}}\sum \limits_{i=1}^{n_{sim}}1\left({p}_i\le \alpha \right)$, where *p*_*i*_ is the *p*-value associated with the null hypothesis tested at *α* = 5%;
- the expectation of $\hat{\theta}$ as $E\left[\hat{\theta}\right]=\frac{1}{n_{sim}}\sum \limits_{i=1}^{n_{sim}}{\hat{\theta}}_i$;
- the bias of $\hat{\theta}$ as $\frac{1}{n_{sim}}\sum \limits_{i=1}^{n_{sim}}{\hat{\theta}}_i-\theta$; and
- the relative bias of $\hat{\theta}$ as $\frac{\left(\frac{1}{n_{sim}}\sum \limits_{i=1}^{n_{sim}}{\hat{\theta}}_i\right)-\theta }{\theta }$.

### Analysis selection strategy

For each simulated trial, two classes of strategy were used to determine whether or not to switch from the base-case analysis (the actual primary analysis specified in the LIPID trial) and select (for reporting) one of the other possible analysis configurations. Strategy A involved switching at random, with 50% probability, from the base-case analysis to a random choice of one of the other 71 possible alternative configurations. Strategy A consequently represented a legitimate approach to making changes to the original design/planned analysis of a RCT *without* knowledge of the association between treatment allocation and outcome.

Strategy B involved selecting (to report) the analysis configuration that yielded the most *extreme* result for a given simulated trial. We operationalised the selection of the ‘most extreme result’ in two ways. The first, Strategy B1, was to select the analysis with the smallest (two-sided) *p*-value reflecting a situation where a ‘statistically significant’ finding is being inappropriately sought to, for example, misguidedly improve the likelihood of results publication. The direction of the treatment effect should have no bearing on the choice of the analysis configuration under Strategy B1 as a choice based on the smallest *p*-value could favour either treatment. The second way, Strategy B2, was to select the analysis with the smallest $\hat{\theta}$—reflecting a situation where a stronger effect is being inappropriately sought to, for example, improve the estimated incremental effectiveness of a new therapy. Strategies B1 and B2 simulated an approach to making illegitimate changes *with* knowledge of the association between treatment allocation and outcome.

### Scenarios evaluated

The change strategies were compared under three scenarios. For Scenario 1, simulations were performed under H0 (*θ* = 0) and all of the 72 possible analysis configurations were available for selection. Strategy A was compared against Strategies B1 and B2.

For Scenario 2, simulations were performed under H0 (*θ* = 0) but constraints were imposed on the selection of possible analysis configurations. Three types of constraint were investigated. The first allowed flexibility away from the base-case only in the selection of the analysis endpoint (three configurations). The second allowed flexibility away from the base-case only in the selection of the analysis set (three configurations). The third allowed flexibility away from the base-case only for covariate specification (eight configurations). The simulations conducted under Scenario 2 thus allowed us to explore the isolated effect of changing only one aspect of the trial while keeping the other aspects fixed.

For Scenario 3, simulations were performed under H1 comparing Strategies A and B. Under H1, a HR of 0.85 was imposed on the CHD death endpoint only (see Additional File 1). For analyses of the simulated data that were restricted to the CHD death endpoint only, *θ* = log(0.85) = − 0.1625. The effect we imposed on CHD death indirectly introduced a treatment effect on the revascularisation and all-cause mortality endpoints because there was some correlation between these events and CHD. Thus, *θ* = − 0.1262 was determined to be the appropriate estimand for analyses of the simulated data with no restriction on the choice of endpoint.

## Results

### Scenario 1

**Fig. 1:**
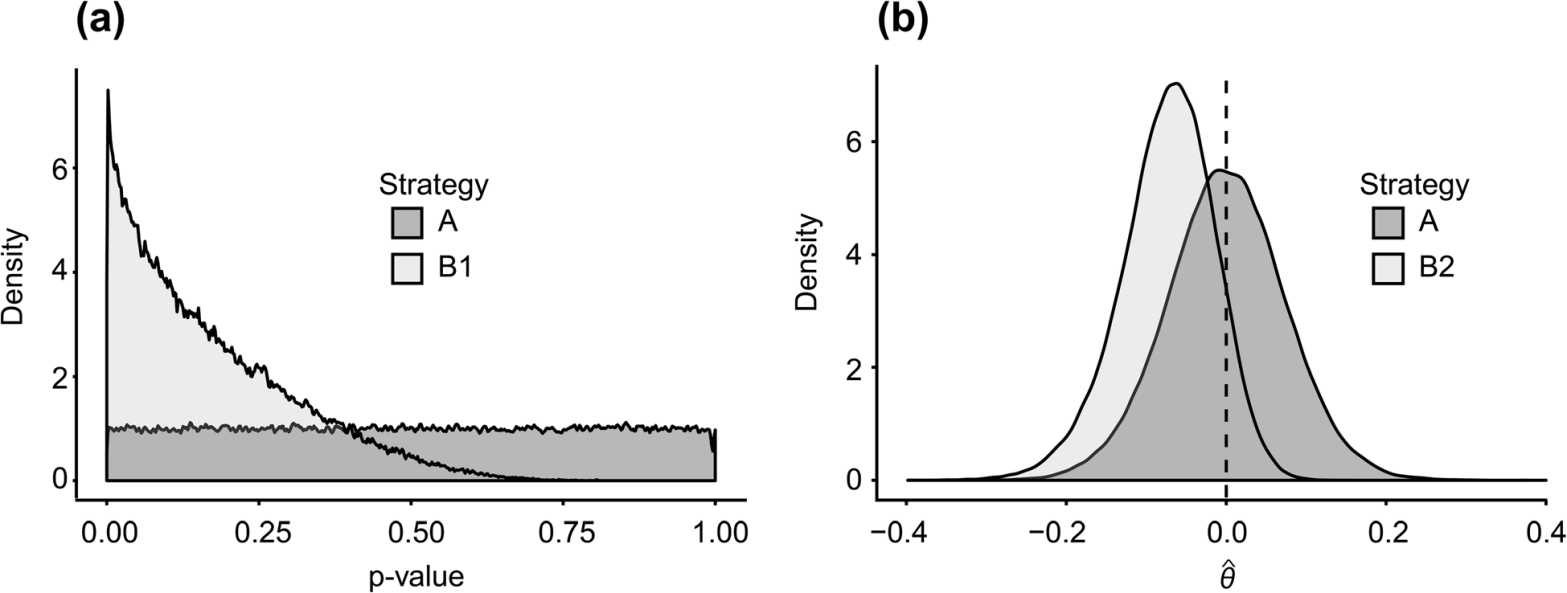
Distribution of *p*-values and $\hat{\theta}$ under H0 (null hypothesis) (Scenario 1)

Under H0, Strategy A produced unbiased results. The *p*-values had a uniform distribution (Fig. 1a), and $\hat{\theta}$ was symmetrically distributed with $E\left[\hat{\theta}\right]$ =0.000 (Fig. 1b). The type 1 error rate of Strategy A was consistent with the 5% significance level targeted (mean: 5.0%, 95% CI: 4.9% to 5.2%). The results for Strategies B1 and B2 were biased under H0. For Strategy B1, *p*-values were right skewed (Fig. 1a), and the type 1 error rate was well above the 5% level (mean: 23.0%, 95% CI: 22.7 to 23.2). For Strategy B2, $\hat{\theta}$ was symmetrically distributed but biased with $E\left[\hat{\theta}\right]$ = − 0.0737 (Fig. 1b).

### Scenario 2

**Fig. 2:**
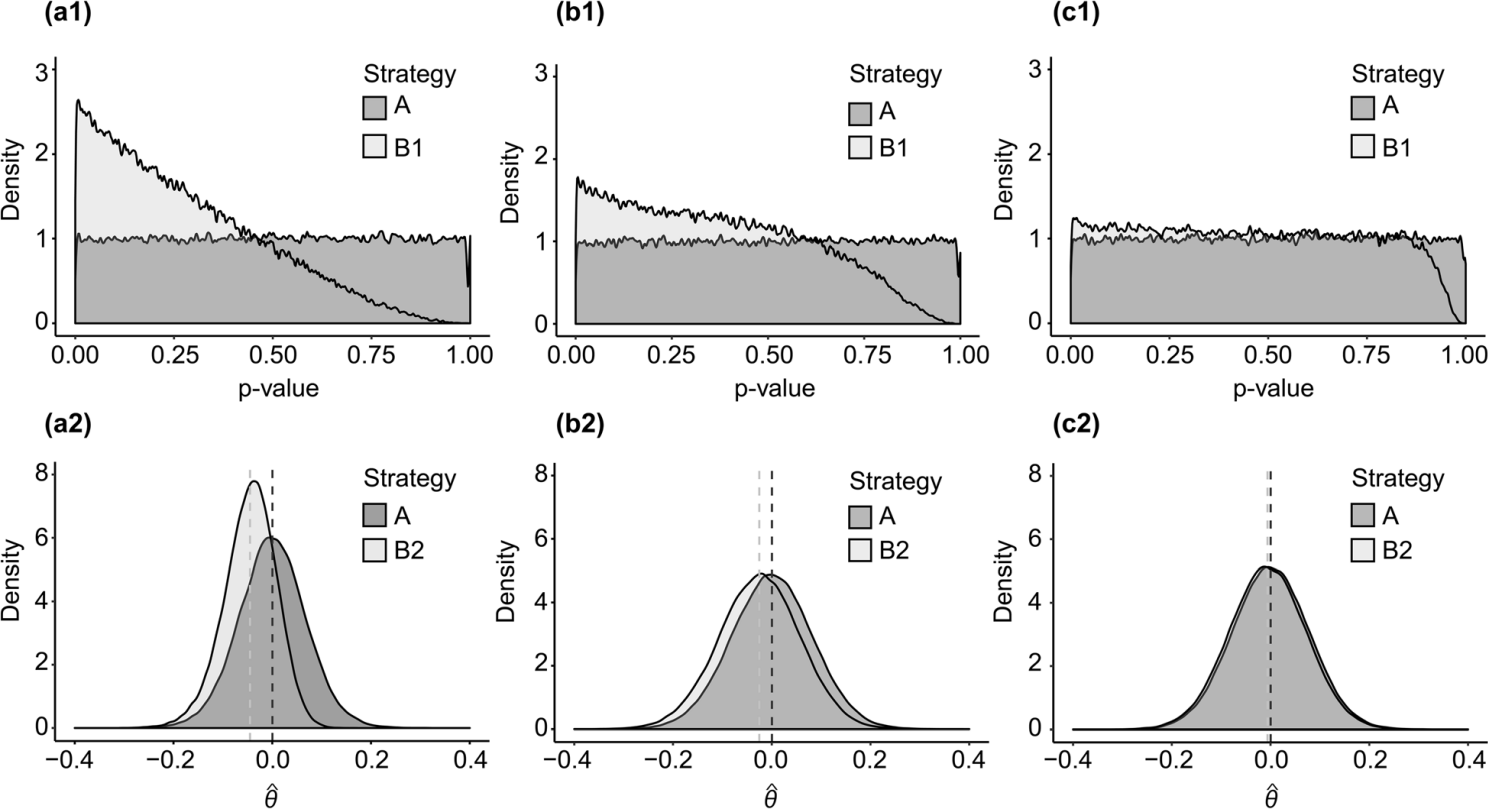
Distribution of *p*-values and $\hat{\theta}$ under H0 (null hypothesis) and flexibility in only one analysis decision category (Scenario 2)

When flexibility in analysis configuration was permitted in only one of the three analysis decision categories under H0, Strategy A again produced unbiased results (in each case $E\left[\hat{\theta}\right]$ =0.000). The bias introduced under Strategies B1 and B2 was greatest when there was freedom to switch only the endpoint (Fig. 2a1, a2), followed by the analysis set composition (Fig. 2b1, b2) and, finally, the covariate specification (Fig. 2c1, c2).

### Scenario 3

**Fig. 3:**
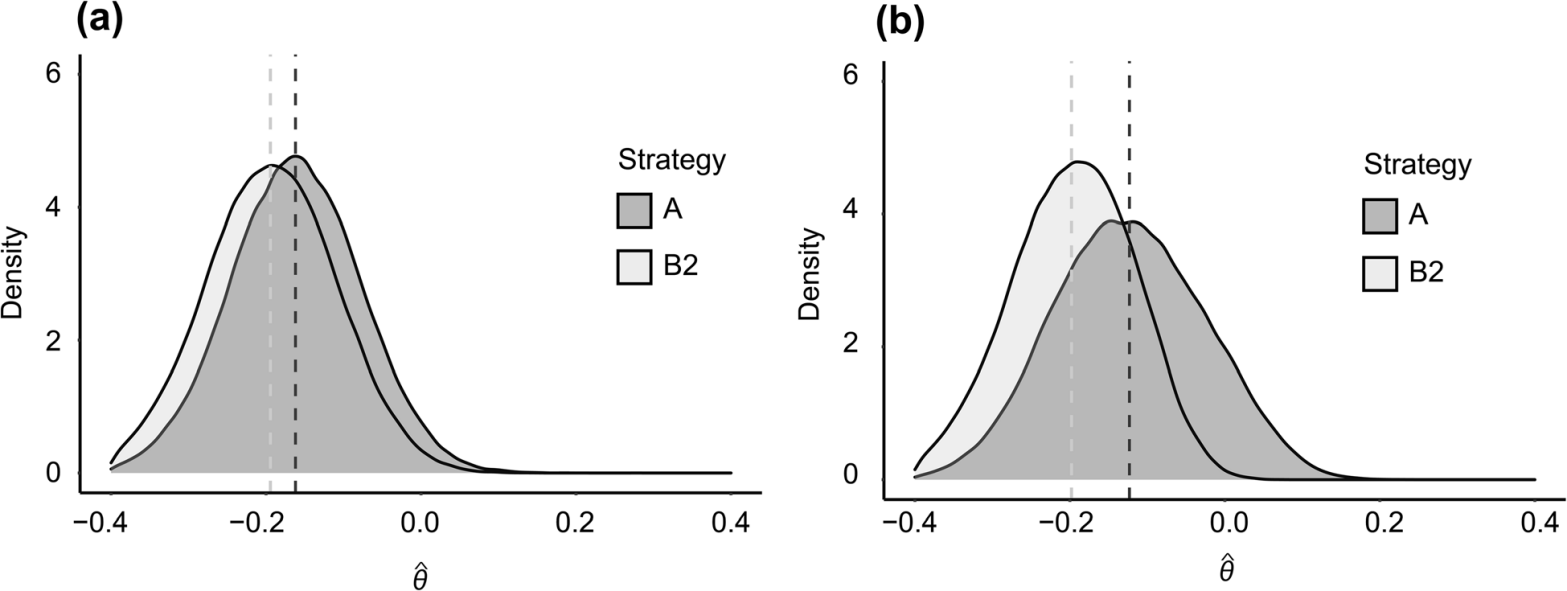
Distribution of $\hat{\theta}$ under H1 (alternative hypothesis) (Scenario 3)

In the initial series run under H1 with forced selection of CHD death as the endpoint but flexibility in the selection of the analysis set and covariate specification (24 configurations), Strategy A was unbiased with $E\left[\hat{\theta}\right]$ = − 0.1620 being very close to the relevant estimand of *θ* = ln(0.85) = − 0.1625 (Fig. 3a). The minimum and maximum $E\left[\hat{\theta}\right]$ value from the subset of analysis configurations that included one or more covariates was − 0.1627 and − 0.1610, respectively. Thus, whilst inclusion of prognostic covariates in a Cox proportional hazards regression model technically changes the measure of the treatment effect from a marginal measure to a conditional measure, the $E\left[\hat{\theta}\right]$ values from the analysis configurations that included covariates were good estimates of *θ*. Under Strategy B2, the density of $\hat{\theta}$ was shifted to the left with $E\left[\hat{\theta}\right]$ = − 0.1944, reflecting a relative bias of 20%.

In the second series run under H1 with no constraint being imposed on analysis configuration (72 configurations), Strategy A was unbiased with $E\left[\hat{\theta}\right]$ = − 0.1232 being close to the relevant estimand *θ* = − 0.1227 (Fig. 3b). Under Strategy B, however, $E\left[\hat{\theta}\right]$ = − 0.1980 reflected a relative bias of 61%.

### All analysis configurations

The distribution of $\hat{\theta}$ across all 72 possible analysis configurations under H0 and H1 is presented as a series of boxplots in Additional file 2.

## Discussion

This study demonstrated how estimates of treatment effect (under H0 and H1) can be sensitive to the approach used to inform decisions to change aspects of an ongoing RCT. The legitimate decision strategy (Strategy A) produced results that were valid (consistent with expectations) under both H0 and H1. This is because all 72 analysis configurations produced $E\left[\hat{\theta}\right]$ values that were consistent with expectations (see Additional file 2). Thus, whilst our decision to specify a 50% switching probability under Strategy A was arbitrary, the switching probability specified does not change the type 1 error probability or $E\left[\hat{\theta}\right]$ under H0. For example, setting the Strategy A switching probability to 100% under H0 produces a type 1 error rate of 0.0504 (95% CI: 0.0491 to 0.0518) and $E\left[\hat{\theta}\right]$ = 0.000.

The type 1 error rate of the illegitimate decision strategies (Strategies B1 and B2) was well in excess of the 5% nominal significance level (reaching 23% with Strategy B1; Fig. 1), and$E\left[\hat{\theta}\right]$ was substantially biased (e.g. a relative bias of 61% was observed with Strategy B2 under H1; Fig. 3).

The three individual analysis decision categories did not contribute equally to the bias under H0 for Strategies B1 and B2. We observed the largest bias being attributable to changing the endpoint, followed by changing the analysis set composition and, finally, by changing the covariate specification. This pattern is not generalisable, however, as it is a product of the unique joint distribution of outcomes and covariates in the LIPID dataset. The result does, however, demonstrate the potential for substantial bias even if flexibility is restricted to only a few configurations.

The set of scenarios and analysis configurations investigated was far from exhaustive. We did not, for example, explore the consequences of switching between an analysis that accounts for ‘centre’ effects and one that does not, even though the LIPID trial was a multicentre trial. Our results nevertheless illustrate the fact that otherwise methodologically appropriate reactive revisions and/or discretionary decisions can be made without introducing a bias, provided they are made completely independently of knowledge of the association between treatment assignment and outcome. This is a more straightforward proposition in cases where the impetus for a change to a double-blinded RCT is based on information external to the trial, but may be more challenging to follow for open-label RCTs and/or when based on information from within the RCT. Our simulation study did not examine the effect of changes that are driven by within-trial factors (e.g. Example 1 in Table 1). These will be the subject of future simulation studies undertaken by our group.

A compelling rationale for changing some aspect of the planned design/analysis of an RCT is potentially more likely to arise in cases where there have been few preliminary data to guide the original plan, and/or when the RCT takes several years to undertake (and new evidence comes to light and/or the clinical landscape changes). Seamless phase II/III trial designs are a good strategy for accommodating the former situation. These include designs that allow for new treatment arms to be added and other (ineffective) treatment arms to be removed, and for decisions to be based on a synthesis of intermediate and definitive endpoint data. Such designs involve statistical concepts that are likely to be less well understood by clinicians undertaking RCTs, and may be challenging to fund via conventional grant schemes for investigator-initiated research (where they will compete for funding with other research proposals with simper statistical designs and more straightforward implementation plans). Traditional RCTs are thus likely to remain a popular design for investigator-initiated research.

Trialists undertaking traditional RCTs should be conscious of the potential for new information to provoke a need to change some aspect of their ongoing trial, and the potential risks to the integrity of the trial if this is not managed appropriately. Appropriate management will involve carefully documenting the rationale and process for making changes to ensure reviewers/readers can make appropriate assessments of the legitimacy of the changes [19].

The SPIRIT statement provides general guidance on what should be included in clinical trial protocols, but it does not provide detailed instruction on the process of documenting changes in protocol amendments once the trial has started [20]. The CONSORT statement provides general guidance on the reporting of changes to the trial outcomes and methods; however, reviews suggest there remains room for improvement [14, 21–23]. A supplement to the current guidelines and checklists would be useful. Ongoing initiatives to promote greater detail in pre-specified endpoint definitions also merit support [23–26].

Gaining access to full trial protocols remains a challenge. Comparisons of planned versus reported clinical trial procedures, methods and analyses have largely involved the review of limited publicly available information from clinical registries [9, 13, 27, 28]. Such limited information is potentially insufficient, given evidence that access to more detailed (not publicly available) information can change reviewers’ opinions [29–34].

Improved access to full protocols, e.g. via existing clinical trial registries, would facilitate the fair appraisal of trial results and deter selective reporting [29, 35]. Counterarguments to such transparency, e.g. that hinge on the need to maintain confidentiality of (commercially sensitive) intellectual property and/or information integral to preserving treatment concealment [29, 36–38], have been challenged by regulatory authorities and others [29, 31, 34, 39]. Even protocols with sensitive information could be prospectively lodged, and the information kept confidential in escrow until study results are disclosed [37]. The implementation of research assessment indicators by academic research institutions, which publicly laud trialists who share their protocols, and journals enforcing policies and building capacity for public sharing, including mandating access to full and complete protocols as opposed to abridged versions, are also pragmatic steps to change.

## Conclusions

This study demonstrated how modifications to the planned design/analysis of an RCT can be made validly, and illustrated how illegitimate changes introduce bias. For example, using illegitimate approaches, we obtained a type 1 error rate under H0 of 23%, and a relative bias of 61% under H1. Studies in other settings have highlighted how flexibility in the analysis approach can inflate the type 1 error [7, 40, 41], but none have focused on the implications of changing the planned design/analysis of RCTs. Our findings thus provide important guidance for trialists (and stakeholders) on how to make a methodologically justifiable change to a planned RCT design/analysis, and highlight the circumstances under which a change can lead to bias. In so doing, our research intends to both encourage trialists to consider how changes are made and documented, and allow clinicians and stakeholders to distinguish between changes that could lead to a systematic bias versus those that do not. This is important for ensuring that methodically unsound changes to RCTs are recognised and avoided, and that RCTs which have undergone methodically sound changes are not incorrectly dismissed as potentially biased.

## Supplementary information

**Additional file 1.** Construction of the simulated trial datasets and analysis configurations.

**Additional file 2.** Boxplots of $\hat{\boldsymbol{\theta}}$ for all 72 analysis configurations under H0 and H1.

## Data Availability

The data that support the findings of this simulation study are available from the LIPID Trial Management Committee; however, restrictions may apply to the availability of these data, due to relevant data protection laws.

## Abbreviations

AMI: Acute myocardial infarction
CHD: Coronary heart disease
CONSORT: Consolidated Standards of Reporting Trials
H0: Null hypothesis
H1: Alternative hypothesis
HR: Hazard ratio
MAMS: Multi-arm multi-stage
RCT: Randomised controlled trial
SPIRIT: Standard Protocol Items: Recommendations for Interventional Trials
UAP: Unstable angina pectoris
