# Supplementary material for: Changes to aspects of ongoing randomised controlled trials with fixed designs
Source: Trials. 2020 Jun 3;21:457. doi: 10.1186/s13063-020-04374-3 (PMC7268339; doi:10.1186/s13063-020-04374-3)
Supplement: Supplementary file 1 — Additional file 1. Construction of the simulated trial datasets and analysis configurations. [file 13063_2020_4374_MOESM1_ESM.docx]

**Additional File 1**

**Construction of Simulated Trial Datasets**

The datasets derived under H0 were constructed by: (1) making a copy of the full LIPID dataset (N=9,014 patients); (2) randomly allocating patients in this copied dataset to one of two groups using a permuted block approach and storing the result; and, (3) repeating Steps 1 and 2 until $i=100,000$ datasets were generated.

Under H1, a HR of 0.85 for time-to-CHD-death, favouring the experimental treatment (group 1) over control (group 0), was specified to reflect a modest but clinically worthwhile and plausible level of effect. The datasets derived under H1 were constructed by: (1) making a copy of the full LIPID dataset (N=9,014 patients); (2) randomly assigning the subset of $j$ patients that had experienced an event to the experimental group ($j_{1}$ patients) versus the control group ($j_{0}$ patients) in a ratio corresponding to HR of 0.85. For rare events such as CHD-death (660 events from 9014 patients), the relative risk ($RR=\frac{p1}{p0}$), where $p1$ is the event risk in group 1 (intervention) and $p0$ is the event risk in group 0 (control) provides a good approximation to the HR, however the exact relationship between the HR and the event risk in each group is $HR=\frac{ln(1-p1)}{ln(1-p0)}$ [1]. Given 660 events from 9014 patients, a 0.855:1.00 allocation ratio was therefore specified such that 0.461 of the 660 events are expected in group 1 and 0.539 are expected in group 0. The remaining $\left( 9,014-j \right)=k$ patients (experiencing no event) were randomly assigned (using a permuted block approach) to the experimental and control groups such that the remaining $\frac{N=9,014}{2}-j_{1}=k_{1}$ patients were allocated to the experimental group, and $\frac{N=9,014}{2}-j_{0}=k_{0}$ patients were allocated to the control group; and, (3) repeating Steps 1 and 2 until $i=100,000$ datasets were generated.

For all simulated trials a non-repeating stream of pseudo-random numbers were generated from a specified seed using the SAS V9.4 implementation of the Mersenne-Twister pseudo-random number generator [2].

**Construction of Analysis Configurations**

The options under the ‘choice of endpoint’ decision included CHD death (660 events), revascularisations (with 1,292 events), and all-cause mortality (with 1,131 events). CHD death was the pre-specified primary endpoint for LIPID while the other two were pre-specified secondary endpoints. We chose revascularisations as an example of a surrogate endpoint (with a greater number of events than CHD death), and all-cause mortality as an example of an endpoint reflecting overall net benefit (of major relevance for decision-making in practice).

The options under the ‘analysis set composition’ decision comprised ‘all randomised patients’, in accordance with the intention-to-treat (ITT) analysis principle, plus two subsets of patients. The first subset was specified in a manner reflecting a decision to exclude a small number of patients on grounds of ineligibility. We arbitrarily chose to apply a criterion excluding patients with poorer kidney function defined by a glomerular filtration rate of <45 millilitres per minute per 1.73m^2^, corresponding to Stage 3b chronic kidney disease or worse. This led to the exclusion of 4% of patients from the ITT analysis set. The second subset of patients was specified in a manner reflecting a decision to maximise the efficacy signal. This was done by focusing on patients hypothesised to more likely to have their risk of an unfavourable outcome modified via a chronic atherosclerotic pathway targeted by statin therapy. We did this by removing patients who had experienced the pre-baseline ‘qualifying’ event (i.e., AMI or UAP) within 9 months of randomisation. This was based on the assumption that the lipid profile modification may have less of an impact during the subacute period following AMI/UAP. This ‘enrichment’ strategy led to the exclusion of 29% of patients from the ITT analysis set.

The options under the ‘covariate specification’ decision comprised three patient characteristics chosen on the basis of their strong prognostic value, previously characterised in a risk-factor modelling analysis by Marschner et al. [3]. At baseline, 369 (4.09%) LIPID patients had experienced a previous stroke, 869 (9.64%) had a history of smoking, and the acute coronary syndrome distribution was: UAP in 3,260 (36.17%), single AMI in 4,719 (52.35%), and multiple AMI in 1,035 (11.48%) patients.

The three decisions were set to the first choice (i.e., Level 1) in the base-case analysis. Thus, in the base-case analysis, the primary endpoint was CHD death, the analysis set comprised all randomised patients, and there was no adjustment for baseline patient characteristics.

The LIPID trial estimated that pravastatin reduced the hazard of CHD death by 24% (i.e. HR = 0.76) using an analysis including all 9014 randomisation patients and a Cox PH model that adjusted for no additional baseline covariates.

**References**

1. VanderWeele, T.J., *Optimal approximate conversions of odds ratios and hazard ratios to risk ratios.* Biometrics, 2019.

2. Matsumoto, M. and T. Nishimura, *Mersenne twister: a 623-dimensionally equidistributed uniform pseudo-random number generator.* ACM Trans. Model. Comput. Simul., 1998. **8**(1): p. 3-30.

3. Marschner, I.C., et al., *Long-term risk stratification for survivors of acute coronary syndromes: Results from the long-term intervention with pravastatin in ischemic disease (LIPID) study.* Journal of the American College of Cardiology, 2001. **38**(1): p. 56-63.
