# Supplementary material for: Changes to aspects of ongoing randomised controlled trials with fixed designs
Source: Trials. 2020 Jun 3;21:457. doi: 10.1186/s13063-020-04374-3 (PMC7268339; doi:10.1186/s13063-020-04374-3)
Supplement: Supplementary file 2 — Additional file 2. Boxplots of $\hat{\boldsymbol{\theta}}$θ^ for all 72 analysis configurations under H0 and H1. [file 13063_2020_4374_MOESM2_ESM.docx]

**Additional File 2**

Figure 4 and Figure 5 show boxplots of $\hat{\boldsymbol{\theta}}$ for all 72 analysis configurations under H0 and H1 respectively. The panels are arranged into columns according choice of endpoint, and into rows according to analysis set. The grey horizontal lines reflect $\boldsymbol{\theta}$ for a given endpoint. Note that $\boldsymbol{\theta}$ is 0 under H0 irrespective of endpoint, but $\boldsymbol{\theta}$ is different under H1 for each endpoint. Within each panel, the 8 possible covariate specifications are presented. The covariate specification did not materially affect $\boldsymbol{E}\left[ \hat{\boldsymbol{\theta}} \right]$. Thus for the LIPID dataset, the conditional and the marginal treatment effect were very similar (this is not a generalisable finding for the Cox proportional hazard model). For the revascularisations endpoint (right hand column) of Figure 5, a slight bias was noticeable under H1. This appeared to be due to a subtle informative censoring effect that was removed when CHD deaths were treated as a competing event in a competing risks analysis of the revascularisations endpoint. For example, a competing risk analysis undertaken on 10,000 trials simulated under H1 produced a $\boldsymbol{E}\left[ \hat{\boldsymbol{\theta}} \right]$ that was within 0.0002 of the relevant $\boldsymbol{\theta}$.

Figure 4: Boxplots of $\hat{\boldsymbol{\theta}}$ Under H0 for All Analysis Configurations

**
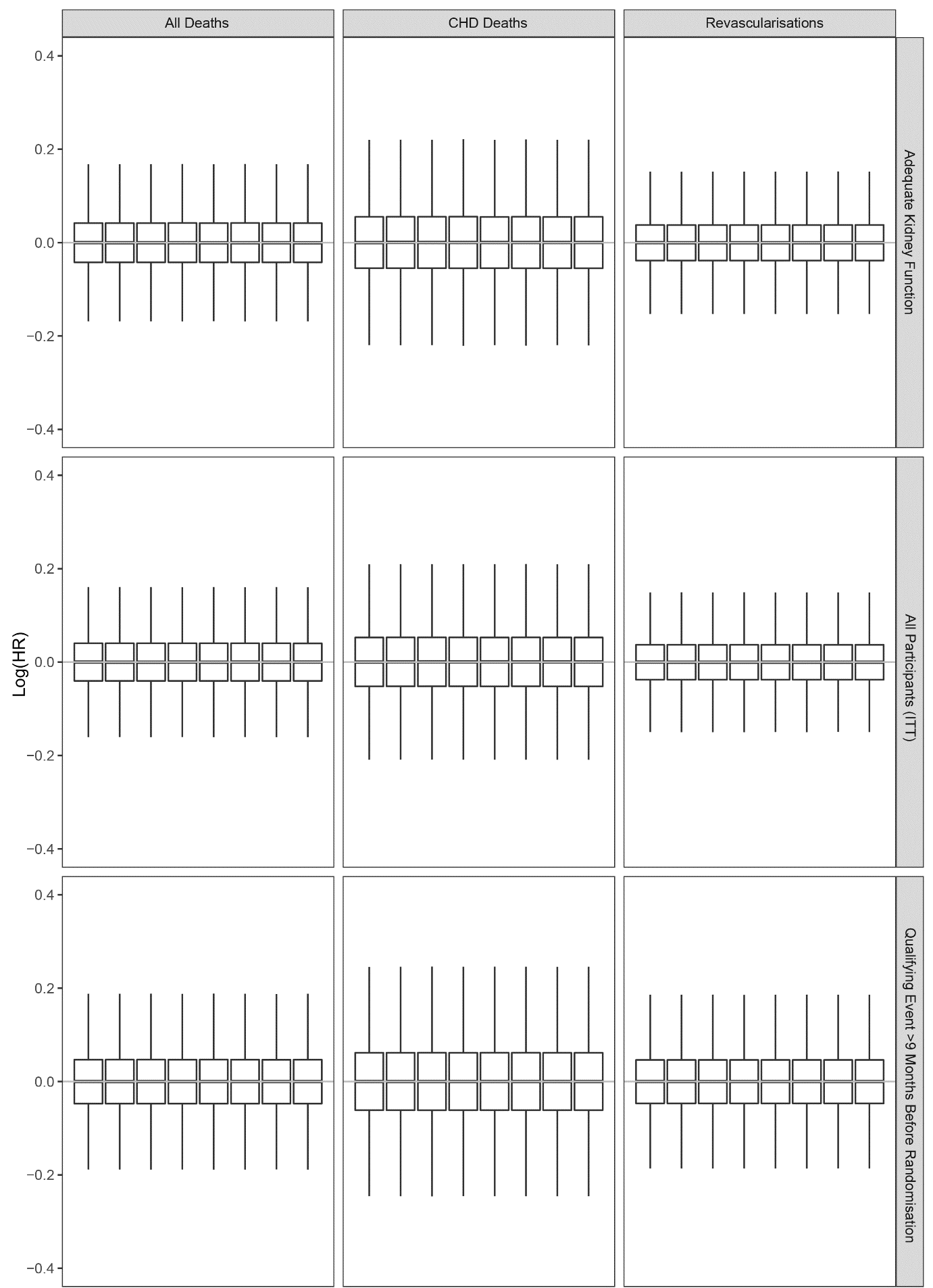
**

Figure 5: Boxplots of $\hat{\boldsymbol{\theta}}$ Under H1 for All Analysis Configurations

**
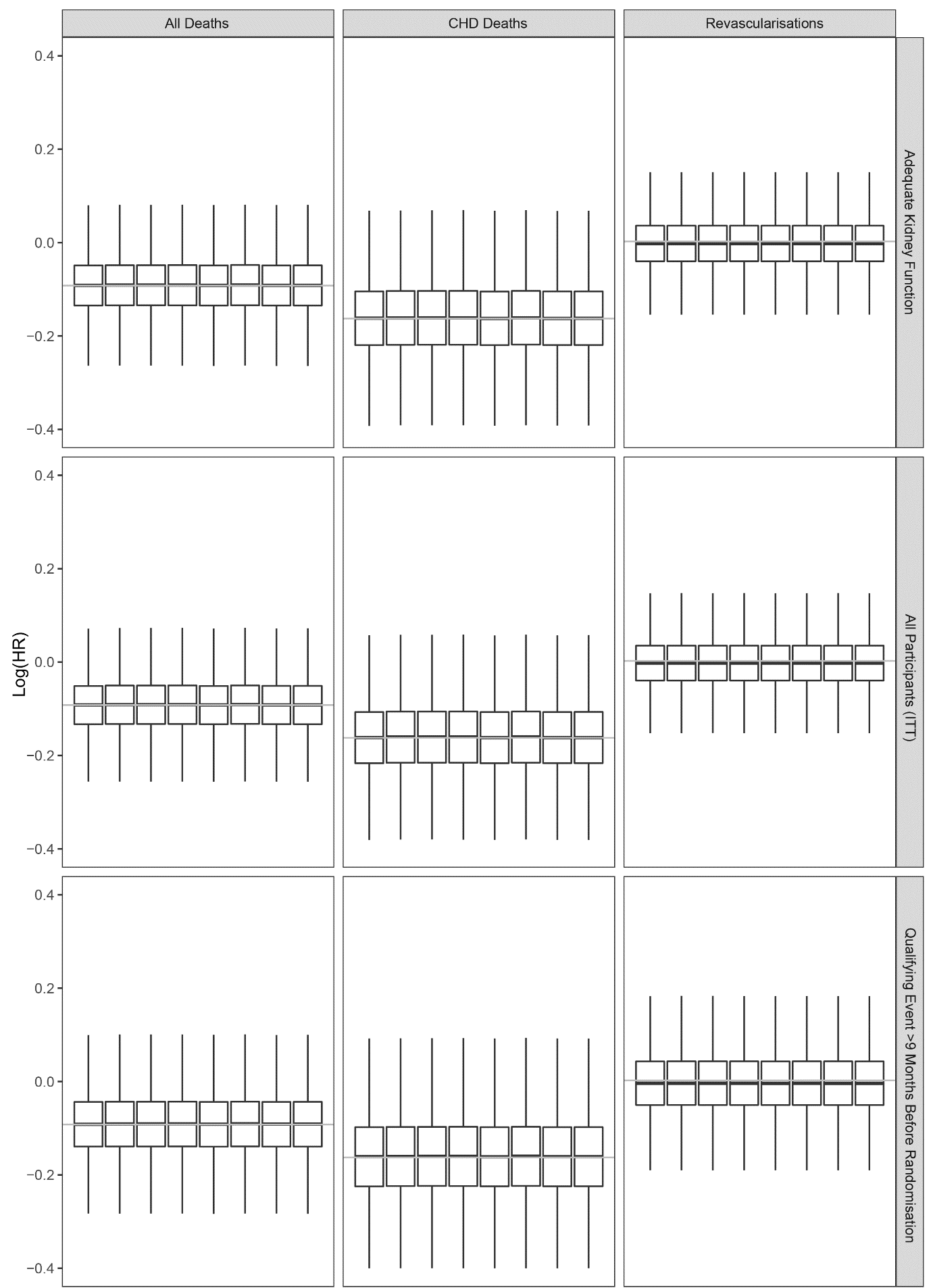
**
